# Preeclampsia: Universal Screening or Universal Prevention for Low and Middle-Income Settings?

**DOI:** 10.1055/s-0041-1729953

**Published:** 2021-05-12

**Authors:** Daniel Lorber Rolnik, Mario Henrique Burlacchini de Carvalho, Guilherme Antonio Rago Lobo, Stefan Verlohren, Liona Poon, Ahmet Baschat, Jon Hyett, Basky Thilaganathan, Emmanuel Bujold, Fabricio da Silva Costa

**Affiliations:** 1 Department of Obstetrics and Gynaecology, Monash University, Melbourne, Australia; 2 Department of Obstetrics and Gynecology, Universidade de São Paulo, São Paulo, SP, Brazil; 3 Department of Obstetrics, Escola Paulista de Medicina, Universidade Federal de São Paulo, São Paulo, SP, Brazil; 4 Department of Obstetrics, Charité Universitätsmedizin, Berlin, Germany; 5 Department of Obstetrics and Gynaecology, The Chinese University of Hong Kong, Hong Kong; 6 The Johns Hopkins Center for Fetal Therapy, Department of Gynecology and Obstetrics, The Johns Hopkins Hospital, Baltimore, United States; 7 RPA Women's and Babies, Royal Prince Alfred Hospital, Sydney, New South Wales, Australia; 8 Fetal Medicine Unit, St George's University Hospitals NHS Foundation Trust, London, United Kingdom; 9 Department of Obstetrics and Gynaecology, Laval University, Quebec, Canada; 10 Maternal Fetal Medicine Unit, Gold Coast University Hospital and School of Medicine, Griffith University, Gold Coast, Australia

Dear Editor,

We read with interest the Clinical Consensus Recommendation about screening and prevention of preeclampsia published by De Oliveira et al. ^1^ The authors recommend that identification of high-risk women should be based on maternal risk factors alone, and that universal treatment (of all pregnant women) with aspirin at a dose of 100 mg should be considered in low- and middle-income countries. In this letter, we express our concerns and disagreement with these strategies.

The association of certain maternal risk factors with an increased risk of preeclampsia development is well known, and several risk scoring systems have been recommended by Obstetric societies around the world, such as the National Institute for Health and Care Excellence (NICE) criteria in the United Kingdom, and the American College of Obstetrician and Gynecologists (ACOG) in the United States. Such scoring systems are based on experts' opinions and low levels of evidence, attribute similar weights to very different risk factors, and perform poorly in the clinical practice. ^1^ Recent large studies ^2^ have shown that such methods fail to identify as high-risk 60% to 70% of women who will later develop preeclampsia. Furthermore, physician compliance with these recommendations is low, with only 20% to 30% of high-risk women receiving aspirin prophylaxis. ^2,3^ On the other hand, combined screening with individual risk calculation by incorporating risk factors, mean arterial blood pressure, uterine artery Doppler studies, and placental growth factor (PlGF) far outperforms risk scoring, identifying as high-risk about three quarters of women who will develop preterm preeclampsia, and 90% of those destined to develop early-onset disease. ^4,5^ In addition, combined screening is more cost-effective, ^6^ and is associated with nearly total physician compliance. ^3^ Although resistance to new technologies is common in Medicine, and translation of research into clinical practice usually takes a long time, ^7^ most components of combined screening, such as the measurement of arterial blood pressure and ultrasound are readily available and widely in use in most settings (even in low/middle-income countries). Simplified versions of the algorithm (for example, without biochemical markers) outperformed clinical history even in middle-income countries such as Brazil, ^8,9^ and could be rapidly implemented with minimal or no increase in cost, and lead to a significant increase in the detection of high-risk women who would benefit from aspirin prophylaxis and likely be missed by risk scoring screening.

As appealing as the suggestion to give aspirin to all pregnant women given its relative safety and low cost may be, a strategy of universal aspirin use has not been properly assessed in adequately-powered prospective studies. ^10^ Pregnant women are naturally resistant to medication use in the absence of convincing medical indication, and such an approach would likely be associated with low adherence to treatment. ^10^ The strong effect of aspirin in the prevention of preeclampsia in high-risk populations ^11^ may not be observed when the treatment is recommended to the entire obstetric population, and side effects will inevitably become more frequent if millions of women are treated. Indeed, data from a previous study on universal aspirin prophylaxis demonstrated no clear treatment benefit, ^12,13^ increased risk of postpartum hemorrhage, ^14^ and other hemorrhagic events, ^12^ as well as low adherence to treatment. ^12^ We argue that early combined prediction with the full or simplified versions of the algorithm for individual risk calculation is feasible in low- and middle-income countries, and should be the preferred method of screening whenever possible, in line with recent recommendations from the International Federation of Gynecology and Obstetrics (FIGO), ^15^ the International Society for the Study of Hypertension in Pregnancy (ISSHP), ^16^ and the International Society of Ultrasound in Obstetrics and Gynecology. ^17^

Conflict of Interests

The authors have no conflict of interests to declare.

**References**

1. De Oliveira LG, Diniz ALD, Prado CAC, Da Cunha Filho EV, De Souza FLP, Korkes HA, et al. Pre-eclampsia: universal screening or universal prevention for low and middle-income settings? Rev Bras Ginecol Obstet 2021;43(1):61–65 10.1055/s-0040-1713803

2. Tan MY, Wright D, Syngelaki A, Akolekar R, Cicero S, Janga D, et al. Comparison of diagnostic accuracy of early screening for pre-eclampsia by NICE guidelines and a method combining maternal factors and biomarkers: results of SPREE. Ultrasound Obstet Gynecol 2018;51(6):743–750 10.1002/uog.19039

3. Guy GP, Leslie K, Diaz Gomez D, Forenc K, Buck E, Khalil A, Thilaganathan B. Implementation of routine first trimester combined screening for pre-eclampsia: a clinical effectiveness study. BJOG 2021;128(2):149–156 10.1111/1471-0528.16361

4. Rolnik DL, Wright D, Poon LCY, Syngelaki A, O'Gorman N, Matallana CP et al. ASPRE trial: performance of screening for preterm pre-eclampsia. Ultrasound Obstet Gynecol 2017;50(4):492–495 10.1002/uog.18816

5. O'Gorman N, Wright D, Poon LC, Rolnik DL, Syngelaki A, Wright A, et al. Accuracy of competing-risks model in screening for pre-eclampsia by maternal factors and biomarkers at 11-13 weeks' gestation. Ultrasound Obstet Gynecol 2017;49(6):751–755 10.1002/uog.17399

6. Park F, Deeming S, Bennett N, Hyett J. Cost effectiveness analysis of a model of first trimester prediction and prevention for preterm preeclampsia against usual care. Ultrasound Obstet Gynecol 2020;•••: 10.1002/uog.22193 [ahead of print]

7. Papageorghiou AT. From evidence to implementation. BJOG 2020;127(10):1173–1174 10.1111/1471-0528.16408

8. Rocha RS, Alves JAG, E Holanda Moura SBM, Araujo Júnior E, Peixoto AB, Santana EFM, et al. Simple approach based on maternal characteristics and mean arterial pressure for the prediction of preeclampsia in the first trimester of pregnancy. J Perinat Med 2017;45(7):843–849 10.1515/jpm-2016-0418

9. Rocha RS, Gurgel Alves JA, E Holanda Moura SBM, Araujo Júnior E, Martins WP, Vasconcelos CTM, et al. Comparison of three algorithms for prediction preeclampsia in the first trimester of pregnancy. Pregnancy Hypertens 2017;10:113–117 10.1016/j.preghy.2017.07.146

10. Cuckle H. Strategies for prescribing aspirin to prevent preeclampsia: a cost-effectiveness analysis. Obstet Gynecol 2020;135(1):217 10.1097/AOG.0000000000003631

11. Rolnik DL, Wright D, Poon LC, O'Gorman N, Syngelaki A, Matallana CP et al. Aspirin versus placebo in pregnancies at high risk for preterm preeclampsia. N Engl J Med 2017;377(7):613–622 10.1056/NEJMoa1704559

12. Subtil D, Goeusse P, Puech F, Lequien P, Biausque S, Breart G, et al; Essai Régional Aspirine Mère-Enfant (ERASME) Collaborative Group. Aspirin (100 mg) used for prevention of pre-eclampsia in nulliparous women: the Essai Régional Aspirine Mère-Enfant study (Part 1). BJOG 2003;110(5):475–484 10.1046/j.1471-0528.2003.02096.x

13. Hoffman MK, Goudar SS, Kodkany BS, Metgud M, Somannavar M, Okitawutshu J, et al; ASPIRIN Study Group. Low-dose aspirin for the prevention of preterm delivery in nulliparous women with a singleton pregnancy (ASPIRIN): a randomised, double-blind, placebo-controlled trial. Lancet 2020;395(10220):285–293 10.1016/S0140-6736(19)32973-3

14. Mone F, Mulcahy C, McParland P, Breathnach F, Downey P, McCormack D, et al. Trial of feasibility and acceptability of routine low-dose aspirin versus Early Screening Test indicated aspirin for pre-eclampsia prevention (TEST study): a multicentre randomised controlled trial. BMJ Open 2018;8(7):e022056 10.1136/bmjopen-2018-022056

15. Poon LC, Shennan A, Hyett JA, Kapur A, Hadar E, Divakar H, et al. The International Federation of Gynecology and Obstetrics (FIGO) initiative on pre-eclampsia: A pragmatic guide for first-trimester screening and prevention. Int J Gynaecol Obstet 2019;145(Suppl 1):1–33 10.1002/ijgo.12802

16. Brown MA, Magee LA, Kenny LC, Karumanchi SA, McCarthy FP, Saito S, et al; International Society for the Study of Hypertension in Pregnancy (ISSHP). Hypertensive disorders of pregnancy: ISSHP classification, diagnosis, and management recommendations for international practice. Hypertension 2018;72(1):24–43 10.1161/HYPERTENSIONAHA.117.10803

17. Sotiriadis A, Hernandez-Andrade E, da Silva Costa F, Ghi T, Glanc P, Khalil A, et al; ISUOG CSC Pre-eclampsia Task Force. ISUOG Practice Guidelines: role of ultrasound in screening for and follow-up of pre-eclampsia. Ultrasound Obstet Gynecol 2019;53(1):7–22 10.1002/uog.20105

**Author's Reply:**

^1^ Department of Gynecology and Obstetrics, Faculdade de Medicina de Botucatu, Universidade Estadual Paulista “Júlio de Mesquita Filho” (UNESP), Botucatu, São Paulo, SP, Brazil

^2^ Department of Obstetrics and Gynecology, Faculdade de Medicina, Universidade Federal de Uberlândia, Uberlândia, Minas Gerais, MG, Brazil

^3^ Department of Gynecology and Obstetrics, Faculdade de Medicina de Ribeirão Preto, Universidade de São Paulo, Ribeirão Preto, São Paulo, SP, Brazil

^4^ Gynecology and Obstetrics Training Center, Escola de Medicina, Pontifícia Universidade Católica do Rio Grande do Sul, Porto Alegre, Rio Grande do Sul, RS, Brazil

^5^ Department of Tocoginecology, Centro Universitário Lusíada, Santos, São Paulo, SP, Brazil

^6^ Department of Obstetrics and Gynecology, Faculdade de Medicina de Sorocaba, Pontifícia Universidade Católica de São Paulo, Sorocaba, São Paulo, SP, Brazil

^7^ Department of Gynecology and Obstetrics, Faculdade de Medicina, Universidade Federal do Rio Grande do Sul, Porto Alegre, Rio Grande do Sul, RS, Brazil

^8^ Department of Gynecology and Obstetrics, Faculdade de Ciências Médicas, Universidade Estadual de Campinas, Campinas, São Paulo, SP, Brazil

^9^ Department of Gynecology and Obstetrics, Faculdade de Medicina, Universidade Federal de Minas Gerais, Belo Horizonte, Minas Gerais, MG, Brazil

^10^ Escola Paulista de Medicina, Universidade Federal de São Paulo, São Paulo, SP, Brazil

Leandro Gustavo De Oliveira, Distrito de Rubião Junior, s/n°, 18618-970, Botucatu, SP, Brazil (e-mail: leandro.gustavo@unesp.br).

Leandro De Oliveira, ^1^ Angélica Lemos Debs Diniz, ^2^ Caio Antônio de Campos Prado, ^3^ Edson Vieira Da Cunha Filho, ^4^ Francisco Lázaro Pereira De Souza, ^5^ Henri Augusto Korkes, ^6^ José Geraldo Ramos, ^7^ Maria Laura Costa, ^8^ Mário Dias Corrêa Junior, ^9^ Nelson Sass, ^10^ Ricardo De Carvalho Cavalli, ^3^ Sérgio Hofmeister De Almeida Martins-Costa, ^7^ José Carlos Peraçoli ^1^

Dr. Leandro De Oliveira's ORCID is 0000-0002-8422-9907

Dr. Angélica Lemos Debs Diniz's ORCID is 0000-0001-8476-6326

Dr. Caio Antônio de Campos Prado's ORCID is 0000-0003-1767-4725

Dr. Edson Vieira Da Cunha Filho's ORCID is 0000-0001-8100-1926

Dr. Francisco Lázaro Pereira De Souza's ORCID is 0000-0003-0761-7700

Dr. Henri Augusto Korkes's ORCID is 0000-0001-5345-3861

Dr. José Geraldo Ramos's ORCID is 0000-0002-3789-885X

Dr. Maria Laura Costa's ORCID is 0000-0001-8280-3234

Dr. Mário Dias Corrêa Junior's ORCID is 0000-0003-4198-0546

Dr. Nelson Sass's ORCID is 0000-0001-7187-1004

Dr. Ricardo De Carvalho Cavalli's ORCID is 0000-0001-5010-4914

Dr. Sérgio Hofmeister De Almeida Martins-Costa's ORCID is 000-0003.0565-974t

Dr. José Carlos Peraçoli's ORCID is 0000-0002-3273-3001

Dear Editor,

We are grateful to the colleague who raised the questions related to our recent publication regarding the use of aspirin and calcium for the prevention of preeclampsia in low- and middle-income countries. 1 Discussions like this are fundamental to raise the importance of the topic, which represents the main cause of maternal death in Brazil. However, we are not sure that the colleague has fully understood our publication, since the costs related to prevention are important, but only part of our text.

We initially expressed great uncertainty regarding the impact of using any algorithms currently demonstrated for the screening of preeclampsia. This is remarkable, since a complex screening model, involving biophysical, biochemical markers, and maternal data recently identified only 0.26% of women who would be diagnosed with preterm preeclampsia in a huge population. 2 The colleague then suggested that protocol adaptations using less complex algorithms according to the economic possibilities of each location could be implemented. However, Prefumo and Farina 3 (2017) raised important considerations about the addition of markers to increase the sensitivity of clinical data in the prediction of preeclampsia. Interestingly, the addition of just one biophysical or biochemical marker does not increase the detection rates, since confidence intervals clearly overlap in most large studies. In the study by O'Gorman et al. 4 (2017), for example, the simple addition of the mean arterial pressure was as efficient as the addition of the uterine artery Doppler or placental growth factor (PlGF) in the prediction of preeclampsia before 32 weeks, with a false-positive rate of 10%. Recently, Sovio and Smith 5 (2019) developed a simple risk score using the same maternal characteristics used for the original algorithm in the Combined Multimarker Screening and Randomized Patient Treatment with Aspirin for Evidence-Based Preeclampsia Prevention (ASPRE) study. 2 5 The area under the receiver operating characteristic (ROC) curve of this simplified score was of 0.846 (95% confidence interval [95%CI]: 0.787–0.906), similar to that of the complete algorithm, which was of 0.854 (95%CI: 0.795–0.914). In addition to the fact that there actually is no efficient algorithm for the prediction of preeclampsia, we speculate that the discussion regarding the use of these complex algorithms may contribute to divert the attention of many clinicians regarding important epidemiological information, leading to low rates of prescription of aspirin and calcium.

The colleague who brings the questions refers elusively about the results regarding the use of aspirin. Therefore, we request caution when studying this issue. When referring to the article published by Hoffman et al. 6 (2020), the colleague should have noticed the enormous impact of this study when the authors demonstrated that nulliparous women from low and middle-income countries who started using aspirin in the period between 6 and 13 weeks and 6 days of pregnancy had a lower incidence of preterm birth and a reduction in perinatal mortality, without adverse effects. 6 Simple and safe, as mentioned by Quinlivan 7 (2020).

Regarding safety, the colleague also raised a question about the increased risk of bleeding among women using aspirin in two studies. The study published by Subtil et al. 8 (2003) demonstrated that there was no difference regarding adverse outcomes between the aspirin (19.5%) and placebo (15.8%) groups (relative risk [RR]: 1.23; 95%CI: 1.06–1.43) as the CI reached the null line. Specifically regarding bleeding-related events, such as epistaxis, metrorrhagia and minimal bleeding from the digestive tract, these were considered minimal by the authors. Additionally, when analyzing the results, we realize that the CI also touched the null line (11.6% versus 9.3%; RR: 1.25; 95%CI: 1.03–1.54).

The study developed by Mone et al. (2018), 9 also cited by the colleague as reporting increased numbers of hemorrhagic events, was a feasibility study with no power for this conclusion. Even so, the colleague should be more cautious, since Mone et al. 9 reported in their safety results that the bleeding cases were spottings unrelated to miscarriages, and that there was only a small number of women who had postpartum bleeding ( *n* = 20 without aspirin; *n* = 26 with aspirin). In conclusion, the authors also pointed out that there was no difference regarding hemoglobin levels < 8 g/dL or need for blood transfusion. Finally, this study demonstrated that even low-risk nulliparous women would be happy to take aspirin, which currently contradicts concerns about the low adherence to this medication. In our interpretation, this adherence relates to the impact of the disease on a specific population, which is huge for low- and middle-income countries. Additionally, if clinicians stopped being confused by those who want to introduce complex algorithms for the prediction of preeclampsia, they would prescribe more aspirin and calcium.

We understand that screenings with innovative technologies such as ultrasound or biological markers are very attractive, but are not feasible in low- and middle-income countries, especially in countries with large territorial areas and huge disparities in terms of resources. The women who actually die in these countries are those with lowest socioeconomic status, and our main efforts must converge to really reach this population to provide basic conditions instead of expensive and useless ones.

Mallampati et al. 10 (2019) developed an elegant study comparing the non-use of aspirin, the use of aspirin by women with positive screening based on biomarkers and Doppler, the use of aspirin based on the presence of clinical markers (USA-Task Force), and the universal use of aspirin in pregnancy. The authors demonstrated that the universal use of aspirin significantly reduced the incidence of preterm preeclampsia when compared with the non-use (805 less cases in 100,000 women), when compared with the use of Doppler and biomarkers (314 less cases in 100,000 women), or when compared with clinical screening (358 less cases in 100,000 women). In addition to improving adherence to prevention, the universal use of aspirin has shown a better cost-benefit ratio, without increasing the incidence of adverse events.

In addition, when the colleague mentions that combined screening does not increase costs, he does not seem to believe that this screening model during the first trimester may even lead to additional concerns, as increased number of antenatal visits, additional tests, and, unfortunately, iatrogenic preterm deliveries due to “altered screening.” All of this using methodologies with little evidence of clinical applicability, as published in the systematic review recently published by De Kat et al. (2019). 11

Finally, when the colleague mentions that combined screening is part of the International Federation of Gynecology and Obstetrics (FIGO) recommendations, he or she refers to the publication by Poon et al. 12 (2019). However, the following recommendations have been displayed on the FIGO web site ( https://www.figo.org/figo-releases-new-guidelines-combat-pre-eclampsia ):

“All pregnant women should be screened for preterm PE [preeclampsia] during early pregnancy in the first-trimester with maternal risk factors and blood pressure. Biomarkers offer a potential for early diagnosis and effective treatment, however, the global community recognizes that further evidence for its applicability in all populations and ethnic groups is required at this stage.

While several studies have evaluated the role of biomarkers or a combination of physical and chemical measurements, further studies are needed to define their additional role in improving early prediction of preterm PE.

FIGO encourages all countries and its member associations to adopt and promote strategies to ensure quality research and eventual consensus.”

Regarding the recommendation by the International Society for the Study of Hypertension in Pregnancy ISSHP also mentioned, this society clearly manifested that first trimester screening could be integrated to health systems with capacity for this, and stressed that cost-effectiveness should be evaluated. 13 Additionally, the ISSHP did not mention anything specifically to low and middle-income settings, and we know that this society is truly aware about all difficulties that such countries have.

Essentially, what often seem to be innovative technologies in the clinical practice may not be cost-effective, may not reach vulnerable populations, and may compromise feasible protocols. At this point, we need to be pragmatic and realistic to support public policies based on the best scientific evidence, to reduce maternal mortality related to preeclampsia in low- and middle-income countries.
